# Supplementary material for: Cross-Sectional Study of University Students’ Attitudes to ‘On Campus’ Delivery of COVID-19, MenACWY and MMR Vaccines and Future-Proofing Vaccine Roll-Out Strategies
Source: Vaccines (Basel). 2022 Aug 10;10(8):1287. doi: 10.3390/vaccines10081287 (PMC9415485; doi:10.3390/vaccines10081287)
Supplement: Supplementary file 1 [file vaccines-10-01287-s001.zip › vaccines-1761686-supplementary.pdf]

Supplementary Tables

Table S1. UniCoVac Questionnaire.

Question	Answers and Sub-Questions
Qu. 2. What is your age group	16-17, 18-21, 21-25, >25, Prefer not answer
Qu. 3. What is your current year of study	Foundation, First, Second, Third, Other, Prefer not to answer
Qu. 4. What is the name of your degree course	Open text box
Qu. 5. Are you an international student	Yes, No, Prefer not answer. 5a. If Yes please state your country of permanent residence (open text box); 5.b. Are you currently living in the UK (Yes, No, Prefer not to answer) 5.b.i. If Yes please state when you arrived in the UK (Before 2020, January-May 2020, July-December 2020, January-May 2021)
Qu.6, What is your gender identity?	Male, Female, Non-binary, Another gender identity, Prefer not to answer
Qu. 7. What is your ethnic group?	Asian/Asian British – Indian, Asian/Asian British – Pakistani, Asian/Asian British – Bangladeshi, Asian/Asian British – Chinese, Asian/Asian British – Any other Asian background, Black/African/Caribbean/ Black British – African, Black/African/Caribbean/ Black British – Caribbean, Black/African/Caribbean/ Black, British – Any other Black/African/Caribbean background, Mixed/multiple ethnic groups - White and Black Caribbean, Mixed/multiple ethnic groups - White and Black African, Mixed/multiple ethnic groups - White and Asian, Mixed/multiple ethnic groups - Any other mixed/multiple ethnic background, White – British, White - Irish White - Gypsy or Irish Travellers, White - Any other white background, Other ethnic group – Arab, Other ethnic group – Any other ethnic background, Prefer not to answer
Qu. 8. Where have you lived during this academic year (tick all that apply)	Oadby student village, Nixon court, Opal court, Brookland Road, Private student housing, Commute from home, Stayed at home, Other, Prefer not to answer
Qu. 9. If you live in the UK, where do you live outside of term time	[please indicate first part of the post-code, eg NG8]
Qu. 10. Have you had, or are you going to have, a vaccination against COVID-19?	I have already had at least one COVID-19 vaccination, I have not had a vaccination but have been told that I will be offered a vaccination in the near future, I have been offered a vaccination but have decided not to have the vaccine, I have not yet been offered a vaccination but intend to have the vaccine when offered, I have not yet been offered a vaccination but have decided not to have a vaccine when offered, Other<br>If selected Other, please specify: (Open text box))<br>For answers 3 or 5, there was a sub-question:- Qu. 10, b. What are your reason(s) for not having the COVID-19 vaccination? Please select all that apply (for individuals answering b. or d. to Qu. 10). i. I have allergies, needle-phobia, am immuno-compromised, or have other clinical reasons not to be vaccinated; ii. I am not convinced that COVID-19 vaccines will be effective; iii. Vaccines may not have been tested thoroughly in all ethnic groups; iv. I have had COVID-19 and therefore do not feel I need the vaccine; v. I do not feel that I personally am at risk from COVID-19; vi. I do not believe in vaccinations in general; vii. It is inconvenient to get a COVID-19 vaccine; viii. None of the above; viii. Prefer not to answer; ix. Other (If you selected Other, please specify (Open text box))<br><br>For answer 1, there was a sub-question: 10.c. Which COVID-19 vaccine did you receive? i. Pfizer-Biontech; ii. Oxford-AstraZeneca; iii. Moderna; iv. Johnson & Johnson; v. Sputnik V; vi. Sinopharm; vii. Sinovac; viii. Unsure; ix. Prefer not to answer; x. Other (If you selected Other, please specify (Open text box))
Qu. 11. How much do you know about COVID-19 vaccines?	indicate on a scale of 1 (low) to 7 (high) or Don't know your level of knowledge of each of these vaccines:- Pfizer-Biontech; Oxford-AstraZeneca; Moderna; Johnson & Johnson; Sputnik V; Sinopharm; Sinovac
Qu. 12. I am concerned about the side-effects of COVID-19 vaccines?	indicate the extent to which you agree or disagree by selecting a number from 1 (Strongly disagree) to 7 (strongly agree) or Don't know: - Oxford-AstraZeneca, Pfizer-Biontech; All COVID-19 vaccines

Qu. 13. Have you received the following vaccines either as a child or adult	a. MMR (measles, mumps and rubella) YES/NO/DON'T KNOW b. MenACWY (meningitis) YES/NO/DON'T KNOW
Qu. 14. Are you aware that all of these vaccines are free in the UK for students?	a. MenACWY/MMR, YES/NO; b, COVID-19 vaccines, YES/NO
Qu. 15. If vaccines were offered on campus, would this affect your decision to be vaccinated?	a. MenACWY/MMR, Definitely increase /Somewhat increase /Neither/ Somewhat decrease/Definitely decrease/Don't know; b. COVID-19, Definitely increase /Somewhat increase /Neither /Somewhat decrease/ Definitely decrease/Don't know
Qu. 16. Which location would be the best for accessing vaccines on campus?	Please select up to three with a ranking of 1 (first) to 3 (third); Registration, Fresher's Fair, Halls of Residence, Student Union. Other main campus building (e.g. Adrian building), Victoria Park Health Centre, Other (If you selected Other, please specify, (open text box))
Qu. 17. How much do you agree with the following statements about vaccinations in general?	indicate the extent to which you agree or disagree by selecting a number from 1 (Strongly disagree) to 7 (strongly agree) or indicate 'Prefer not to answer' :-<br>I can rely on vaccines to stop serious infectious diseases; Although most vaccines appear to be safe, there may be problems that we have not yet discovered; Authorities promote vaccination for financial gain, not for people's health; A high level of vaccine coverage (>95%) is required to prevent spread of infectious diseases; Being exposed to diseases naturally is safer for the immune system than being exposed through vaccination
Qu. 18. Do you think that you currently have or have had COVID-19?	No; Unsure; Yes, my own suspicions; Yes, suspected by a doctor but not tested; Yes, confirmed by a positive test; Prefer not to answer. If answer 5 was selected then the following question appeared:- 18.a. Which type of test were/are you positive (PCR, Lateral flow, Both, Unsure, Other). 18a.i. If you selected Other, please specify: (open text box)
Qu. 19. How concerned are you that you will get COVID-19 and require hospitalisation?	Not at all concerned; A little concerned; Quite concerned; Very concerned; Prefer not to answer
Qu. 20. How concerned are you that you might unknowingly spread COVID-19 to others?	Not at all concerned; A little concerned; Quite concerned; Very concerned; Prefer not to answer
Qu. 21. Do you personally know anyone who has died from COVID-19?	(Please select all that apply)<br>Yes, family member(s); Yes, friend(s); Yes, someone else; No; Prefer not to answer
Qu. 22. Where do you get information about COVID-19? (Please select all that apply)	Other students; Friends, family, neighbours; Television, radio, newspapers or magazines; University email or poster; Government or NHS posters, adverts, leaflets or website; Twitter; Tiktok; Other social media (e.g. Facebook, Instagram); World Health Organisation website; Other websites; Scientific journals; Not applicable; Prefer not to answer
Qu. 23. Have you experienced any harassment as a result of COVID-19? **This could take the form of verbal abuse, intimidatory or threatening behaviour, receiving hostile messages online, unwanted sexual contact or physical attacks.**	Yes, No, Prefer not to answer If a Yes answer was provided the following questions appeared:- 24. What kind of harassment have you experienced as a result of COVID-19? (Verbally abused; Threatened or intimidated in person; Threatened or intimidated online; Deliberate damage to property; Physically attacked; Sexually harassed or assaulted); 25. What do you think was the main reason for the harassment you have experienced as a result of COVID-19? **Please note that you can be targeted for your perceived association to a group, whether or not you actually identify as a member of it. (**Your age; Your national origins; Your race; Your language or accent; Your religion; An aspect of your physical appearance; Other (If you selected Other, please specify: (Open text box)); Prefer not to answer
Qu. 26. For each of the following statements, indicate the extent to which you agree or disagree by selecting a number from 1 (Strongly disagree) to 7 (strongly agree) or Prefer not to answer.	i. My life is determined by my own actions; ii. I am usually able to protect my personal interests; iii. I can pretty much determine what will happen in my life; iv. To a great extent, my life is controlled by accidental happenings; v. Often there is no chance of protecting my personal interest from bad luck happenings; vi. When I get what I want, it's usually because I'm lucky; vii. People like myself have very little chance of protecting our personal interests where they conflict with those of strong pressure groups; viii. My life is chiefly controlled by

	powerful others; ix. I feel like what happens in my life is mostly determined by powerful people; x. If someone is meant to have a serious disease, they will get that disease; xi. My health is determined by fate; xii. My health is determined by something greater than myself; xiii. I will stay healthy if I am lucky; xiv. I like having a clear and structured mode of life; xv. I would quickly become impatient and irritated if I could not find a solution to a problem immediately; xvi. Genes are more important than one's own behaviour in determining one's health
Qu. 27. How satisfied or dissatisfied are you with the support you received from the University of Leicester during the COVID-19 pandemic	Very satisfied; Moderately satisfied; Neither; Moderately dissatisfied; Very dissatisfied; Prefer not to answer
Qu. 28. Is there anything specific that the University of Leicester could do to mitigate the impact on students of any future COVID-19 waves? *	Open text box
Qu. 29. Would you be willing to be contacted by the project team to discuss your experiences in more detail?	Yes, No

* This question was not compulsory.

Table S2. Weighting values for the weighted multivariate analysis.

Ethnic_group	Gender	Weight
White	Female	1.0963362
White	Male	1.7224416
White	Other	0.0782102
Asian	Female	0.4013974
Asian	Male	0.6306310
Asian	Other	0.0286348
Black	Female	0.8557532
Black	Male	1.3444643
Black	Other	0.0610475
Prefer not to say	Female	1.0961934
Prefer not to say	Male	1.7222171
Other	Female	0.6639342
Other	Male	1.0430996
Other	Other	0.0473636

Table S3. Comparison of ethnicity demographics of UniCoVac questionnaire participants to University of Leicester and UK universities

Ethnic group	Survey	University of Leicester[1]	UK universities[1]
White	479 (58%)	4,798 (41%)	1,444,450 (74%)
Asian	203 (25%)	4,055 (34%)	226,595 (12%)
Black	69 (8%)	1,552 (13%)	152,420 (8%)
Other	65 (8%)	794 (7%)	118,250 (8%)
(Unknown)	11 (1%)	540 (5%)	33,660 (2%)

[1]Ethnicity distribution for UniCoVac survey sample is distinct from both the University of Leicester and UK Universities (Chi-squared, 3 d.f., $p < 0.00001$)

Table S4. Comparison of gender demographics of UniCoVac questionnaire participants to University of Leicester and UK universities

Gender	Survey	University of Leicester[1]	UK universities[1]
Female	548 (66%)	6,109 (52%)	1,440,815 (57%)
Male	255 (31%)	5,630 (48%)	1,087,710 (43%)
Other	18 (2%)	-	3,865 (0.2%)
Unknown	6 (1%)	-	-

[1]Gender distribution for survey sample is distinct from both the University of Leicester and UK Universities (Chi-squared, 1 d.f., $p < 0.00001$), with an over-representation of female students

Table S5. Comparison of demographics for Year groups between UniCoVac questionnaire participants and University of Leicester undergraduate population

Year of study	Survey	University[1]
Foundation	16 (2%)	550 (5%)
First	269 (33%)	3760 (33%)
Second	217 (26%)	3298 (29%)
Third	234 (28%)	2951 (26%)
Other	91 (11%)	741 (7%)

[1]Chi-squared test: p = 0.22

Table S6. Classification of questionnaire respondents for vaccine hesitancy (dichotomous)[1].

Classification and Response to Survey Qu. 10	UK/International Student Status				
	UK student	UK-based international student	Non-UK international student	No answer	Total
**Not hesitant**<br>I have already had at least one COVID-19 vaccination	475 (65%)	26 (54%)	25 (66%)	1 (14%)	527 (64%)
**Hesitant**<br>I have been offered a vaccination but have decided not to have the vaccine	18 (2.5%)	3 (6.2%)	0 (0%)	1 (14%)	22 (2.7%)
**Unknown**<br>I have not had a vaccination but have been told that I will be offered a vaccination in the near future	49 (6.7%)	2 (4.2%)	2 (5.3%)	1 (14%)	54 (6.5%)
**Hesitant**<br>I have not yet been offered a vaccination but have decided not to have a vaccine when offered	13 (1.8%)	2 (4.2%)	1 (2.6%)	2 (29%)	18 (2.2%)
**Not hesitant**<br>I have not yet been offered a vaccination but intend to have the vaccine when offered	168 (23%)	15 (31%)	9 (24%)	2 (29%)	194 (23%)
**Unknown**<br>Other	11 (1.5%)	0 (0%)	1 (2.6%)	0 (0%)	12 (1.5%)
Total	734 (100%)	48 (100%)	38 (100%)	7 (100%)	827 (100%)

[1]Students were classed as vaccine hesitant / not hesitant or unknown according to their answers to the question "10. Have you had, or are you going to have, a vaccination against COVID-19 (Please select one)?".

Table S7. Univariable analysis of attitudes to on campus delivery of MMR and MenACWY vaccines

Characteristic	On-campus COVID19 vaccinations				On-campus MMR vaccinations			
	Ambivalent (n = 320)[1]	In Favour (n = 507)[1]	p-value[2]	q-value[3]	Ambivalent (n = 355)[1]	In Favour (n = 472)[1]	p-value[2]	q-value[3]
COVID19 vaccine hesitancy			**<0.001**	**<0.001**			**<0.001**	**0.002**
0	260 (36%)	461 (64%)			292 (40%)	429 (60%)		
1	30 (75%)	10 (25%)			28 (70%)	12 (30%)		
Unknown	30	36			35	31		
Ethnic group			>0.9	>0.9			0.7	0.7
White	185 (39%)	294 (61%)			196 (41%)	283 (59%)		
Asian	78 (38%)	125 (62%)			90 (44%)	113 (56%)		
Black	28 (41%)	41 (59%)			33 (48%)	36 (52%)		
Prefer not to say	3 (27%)	8 (73%)			5 (45%)	6 (55%)		
Other	26 (40%)	39 (60%)			31 (48%)	34 (52%)		
Gender			0.8	>0.9			0.3	0.4
Female	209 (38%)	339 (62%)			231 (42%)	317 (58%)		
Male	102 (40%)	153 (60%)			115 (45%)	140 (55%)		
Unknown	3 (50%)	3 (50%)			4 (67%)	2 (33%)		
Other	6 (33%)	12 (67%)			5 (28%)	13 (72%)		
Course studied			0.5	0.7			0.4	0.4
Humanities, Law and Social Science	127 (36%)	222 (64%)			148 (42%)	201 (58%)		
Natural and Life Sciences	123 (41%)	179 (59%)			138 (46%)	164 (54%)		
Medicine and allied	70 (40%)	106 (60%)			69 (39%)	107 (61%)		
Home / international student			0.080	0.2			0.2	0.3
UK student	287 (39%)	447 (61%)			318 (43%)	416 (57%)		
UK-based international student	12 (25%)	36 (75%)			15 (31%)	33 (69%)		
Non-UK international student	18 (47%)	20 (53%)			19 (50%)	19 (50%)		
Unknown	3	4			3	4		
Non-term residence			0.090	0.2			0.3	0.4
Local	74 (46%)	86 (54%)			77 (48%)	83 (52%)		
National	199 (37%)	335 (63%)			224 (42%)	310 (58%)		
International	30 (35%)	56 (65%)			34 (40%)	52 (60%)		
Unknown	17	30			20	27		
Age group			0.092	0.2			0.4	0.4
22+	126 (42%)	171 (58%)			133 (45%)	164 (55%)		
<= 21	193 (36%)	336 (64%)			221 (42%)	308 (58%)		
Unknown	1	0			1	0		
Experience of harassment			**0.016**	0.093			**0.023**	0.088
No	299 (40%)	453 (60%)			330 (44%)	422 (56%)		
Yes	15 (24%)	47 (76%)			18 (29%)	44 (71%)		
Unknown	6	7			7	6		
COVID-19 related death in known contact			0.4	0.6			0.4	0.4
No	177 (37%)	298 (63%)			196 (41%)	279 (59%)		
Yes	139 (40%)	207 (60%)			154 (45%)	192 (55%)		

Characteristic	On-campus COVID19 vaccinations				On-campus MMR vaccinations			
	Ambivalent (n = 320)[1]	In Favour (n = 507)[1]	p-value[2]	q-value[3]	Ambivalent (n = 355)[1]	In Favour (n = 472)[1]	p-value[2]	q-value[3]
Unknown	4	2			5	1		
Concern of vaccine side-effects (Oxford/AstraZeneca)			0.9	>0.9			0.12	0.3
1	71 (42%)	100 (58%)			75 (44%)	96 (56%)		
2	63 (41%)	90 (59%)			64 (42%)	89 (58%)		
3	27 (27%)	72 (73%)			29 (29%)	70 (71%)		
4	38 (36%)	67 (64%)			36 (34%)	69 (66%)		
5	36 (35%)	68 (65%)			46 (44%)	58 (56%)		
6	31 (41%)	44 (59%)			38 (51%)	37 (49%)		
7	44 (47%)	50 (53%)			51 (54%)	43 (46%)		
Unknown	10	16			16	10		
Concern over hospitalisation			0.7	0.8			>0.9	>0.9
0	160 (41%)	232 (59%)			169 (43%)	223 (57%)		
1	125 (37%)	214 (63%)			144 (42%)	195 (58%)		
2	21 (35%)	39 (65%)			26 (43%)	34 (57%)		
3	12 (36%)	21 (64%)			14 (42%)	19 (58%)		
Unknown	2	1			2	1		
Concern over spreading COVID-19 to others			0.4	0.6			0.2	0.3
0	35 (43%)	47 (57%)			38 (46%)	44 (54%)		
1	95 (42%)	131 (58%)			109 (48%)	117 (52%)		
2	106 (36%)	191 (64%)			118 (40%)	179 (60%)		
3	81 (38%)	135 (62%)			87 (40%)	129 (60%)		
Unknown	3	3			3	3		
VAX score	0.61 (0.50, 0.71)	0.61 (0.50, 0.71)	0.6	0.8	0.61 (0.50, 0.71)	0.61 (0.50, 0.71)	**0.039**	0.12
Unknown	17	12			17	12		
Year of study			0.4	0.6			0.4	0.4
0	5 (31%)	11 (69%)			5 (31%)	11 (69%)		
1	111 (41%)	158 (59%)			124 (46%)	145 (54%)		
2	73 (34%)	144 (66%)			83 (38%)	134 (62%)		
3	93 (40%)	141 (60%)			104 (44%)	130 (56%)		
4	38 (42%)	53 (58%)			39 (43%)	52 (57%)		
Term-time residence			**<0.001**	**0.004**			**<0.001**	**<0.001**
Home	93 (54%)	78 (46%)			100 (58%)	71 (42%)		
Halls	48 (32%)	104 (68%)			58 (38%)	94 (62%)		
Private	118 (35%)	215 (65%)			128 (38%)	205 (62%)		
Prefer not to answer	6 (100%)	0 (0%)			6 (100%)	0 (0%)		
Other	55 (33%)	110 (67%)			63 (38%)	102 (62%)		
Self-determination score	80 (72, 87)	79 (71, 88)	0.4	0.6	80 (71, 88)	79 (72, 87)	0.3	0.4
Unknown	31	55			44	42		
MMR vaccination at childhood[4]			**0.007**	0.064			**0.002**	**0.010**
Yes	286 (40%)	427 (60%)			308 (43%)	405 (57%)		
No	11 (52%)	10 (48%)			16 (76%)	5 (24%)		

Characteristic	On-campus COVID19 vaccinations Ambivalent (n = 320)[1]	In Favour (n = 507)[1]	p-value[2]	q-value[3]	On-campus MMR vaccinations Ambivalent (n = 355)[1]	In Favour (n = 472)[1]	p-value[2]	q-value[3]
Don't Know	23 (25%)	70 (75%)			31 (33%)	62 (67%)		
MenACWY vaccination at childhood[4]			0.061	0.2			0.078	0.2
Yes	230 (40%)	345 (60%)			260 (45%)	315 (55%)		
No	18 (51%)	17 (49%)			16 (46%)	19 (54%)		
Don't Know	72 (33%)	145 (67%)			79 (36%)	138 (64%)		

[1]n (%); Median (IQR)
[2]Pearson's Chi-squared test; Fisher's exact test; Wilcoxon rank sum test
[3]False discovery rate correction for multiple testing
[4]Response groups: In favour = Definitely or somewhat increase, Ambivalent = neither, definitely or somewhat decrease
[5]ND, no data

Table S8. Multivariate analysis of attitudes to on campus delivery of MMR and MenACWY vaccines

	On-campus COVID-19 (weighted)					On-campus MenACWY / MMR (weighted)				
Characteristic	N	OR[1]	95% CI[1]	p-value	q-value[2]	N	OR[1]	95% CI[1]	p-value	q-value[2]
COVID19 vaccine hesitancy										
0	411	—	—			411	—	—		
1	13	0.08	0.02, 0.44	**0.003**	**0.030**	13	0.51	0.12, 2.22	0.4	0.6
Gender										
Female	280	—	—			280	—	—		
Male	142	0.93	0.61, 1.41	0.7	0.9	142	0.87	0.58, 1.32	0.5	0.6
Ethnic group										
White	257	—	—			257	—	—		
Asian	104	1.30	0.78, 2.17	0.3	0.5	104	1.20	0.74, 1.95	0.5	0.6
Black	35	2.80	1.12, 7.03	**0.028**	0.11	35	1.67	0.72, 3.86	0.2	0.4
Other	35	1.01	0.51, 2.02	>0.9	>0.9	35	0.79	0.40, 1.57	0.5	0.6
Age group										
22+	173	—	—			173	—	—		
<= 21	271	1.73	1.09, 2.76	**0.021**	0.11	271	1.44	0.90, 2.31	0.12	0.3
Course studied										
Humanities, Law and Social Science	166	—	—			166	—	—		
Natural and Life Sciences	163	0.64	0.40, 1.02	0.060	0.2	163	0.66	0.41, 1.04	0.072	0.2
Medicine and allied	110	0.88	0.51, 1.53	0.7	0.9	110	0.97	0.57, 1.65	>0.9	>0.9
Non-term residence										
Local	80	—	—			80	—	—		
National	304	0.91	0.49, 1.68	0.8	0.9	304	0.95	0.52, 1.74	0.9	>0.9
International	49	0.68	0.29, 1.60	0.4	0.6	49	0.75	0.33, 1.71	0.5	0.6
Experience of harassment										
No	407	—	—			407	—	—		
Yes	36	2.03	0.91, 4.51	0.083	0.2	36	2.23	0.98, 5.10	0.057	0.2
COVID-19 related death in known contact										
No	245	—	—			245	—	—		
Yes	183	0.79	0.52, 1.18	0.2	0.5	183	0.74	0.50, 1.09	0.13	0.3
Concern of vaccine side-effects (Oxford/AstraZeneca)	599	0.94	0.85, 1.05	0.3	0.5	599	0.88	0.79, 0.98	**0.024**	0.14
Concern over hospitalisation	599	1.07	0.79, 1.44	0.7	0.9	599	0.94	0.71, 1.24	0.7	0.7
Concern over spreading COVID-19 to others	599	1.06	0.84, 1.33	0.6	0.9	599	1.18	0.94, 1.48	0.2	0.3
VAX score	599	0.38	0.08, 1.66	0.2	0.4	599	0.36	0.08, 1.54	0.2	0.3
Year of study	599	1.21	0.95, 1.52	0.12	0.3	599	1.12	0.88, 1.41	0.4	0.6
Term-time residence										
Home	86	—	—			86	—	—		
Halls	74	3.50	1.61, 7.61	**0.002**	**0.021**	74	2.32	1.09, 4.94	**0.030**	0.14
Private	173	2.59	1.34, 4.99	**0.005**	**0.030**	173	2.36	1.24, 4.46	**0.009**	0.11

	On-campus COVID-19 (weighted)					On-campus MenACWY / MMR (weighted)				
Characteristic	N	OR[1]	95% CI[1]	p-value	q-value[2]	N	OR[1]	95% CI[1]	p-value	q-value[2]
Prefer not to answer	1	0.00	0.00, 0.00	**<0.001**	**<0.001**	1	0.00	0.00, 0.00	**<0.001**	**<0.001**
Other	79	2.15	1.08, 4.29	**0.029**	0.11	79	2.10	1.06, 4.16	**0.032**	0.14
Self-determination score	599	1.00	0.98, 1.02	>0.9	>0.9	599	0.99	0.98, 1.01	0.4	0.6
MMR vaccination at childhood										
Yes	398	—	—			398	—	—		
No	6	0.64	0.06, 7.16	0.7	0.9	6	0.15	0.03, 0.82	**0.028**	0.14
Don't Know	45	2.59	1.03, 6.55	**0.044**	0.14	45	0.98	0.43, 2.24	>0.9	>0.9
MenACWR vaccination at childhood										
Yes	310	—	—			310	—	—		
No	14	1.11	0.28, 4.41	0.9	>0.9	14	3.03	0.72, 12.7	0.13	0.3
Don't Know	115	1.03	0.62, 1.72	>0.9	>0.9	115	1.76	1.03, 2.98	**0.037**	0.14

[1]OR = Odds Ratio, CI = Confidence Interval
[2]False discovery rate correction for multiple testing

Table S9. Reasons for hesitancy of vaccine hesitant individuals.

Response	Number Individuals	Percentage of Individuals
1. I have allergies, needle-phobia, am immuno-compromised, or have other clinical reasons not to be vaccinated	4	10%
2. I am not convinced that COVID-19 vaccines will be effective	17	42.5%
3. Vaccines may not have been tested thoroughly in all ethnic groups	15	37.5%
4. I have had COVID-19 and therefore do not feel I need the vaccine	4	10%
5. I do not feel that I personally am at risk from COVID-19	13	32.5%
6. I do not believe in vaccinations in general	3	7.5%
7. It is inconvenient to get a COVID-19 vaccine	8	20%
8. None of the above	5	12.5%
9. Prefer not to answer	2	5%
10. Other	8	20%

[1]Individuals who indicated that they would not take up a COVID-19 vaccine (n=40) were asked to ask a sub-question; in the sub-question they could select multiple responses from 10 statements as their reason for not having a COVID-19 vaccination

Supplementary Figures

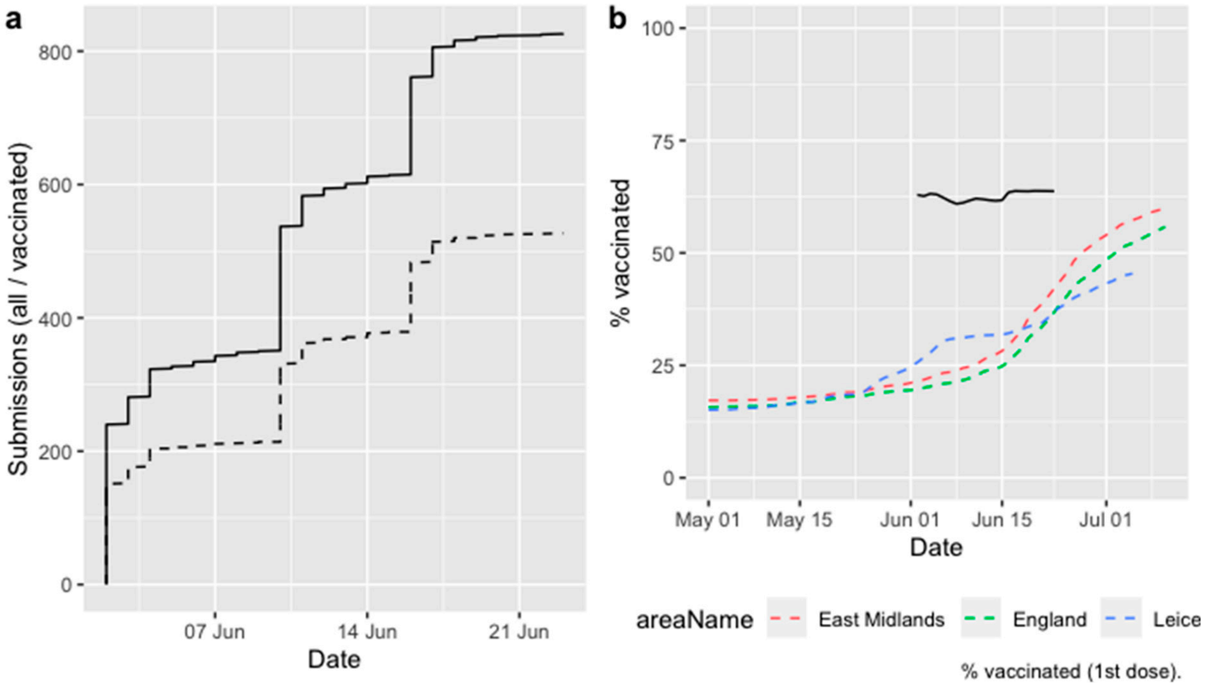

Figure S1. Comparison of the vaccination levels of UniCoVac participants to comparator populations. A total of 527 participants (63.7%) indicated that they had had 'at least one dose of a COVID-19 vaccine' in response to question 10 of the UniCoVac questionnaire. This data was analysed relative to the time of submission of the questionnaire to determine whether the proportion of immunised individuals increased during the study and in comparisons to local and countrywide rates for an age-matched population (note that 94% of UniCoVac participants were in the 18-25 age bracket). We note that surge vaccinations were performed in Leicester between 25/05/2021 and 6/06/2021 with 21,500 vaccinations being administered in multiple locations including De Montfort Hall which is adjacent to the University of Leicester campus. Graph **a** shows the accumulated submissions (solid line) for the three weeks that the survey was open and the accumulated number of submissions from vaccinated individuals (dashed line). The submission spikes on the 1st, 10th and 16th June equate to days when the questionnaire was sent out to students (indicating that most students answer the survey within a few hours of receipt of the email). There is no substantial difference during the collection period indicating that any effect of the surge vaccinations on uptake occurred prior to but not during the study period. Graph **b** shows the % of vaccinated individuals for the UniCoVac participants (solid black line) and 18-24 year olds in Leicester (dashed blue line), East Midlands (dashed red line) and England (dashed green line). A statistical comparison was performed relative to the Leicester cohort as this cohort has the highest uptake and because a high proportion of University of Leicester students would have been able to take advantage of surge vaccinations in Leicester.

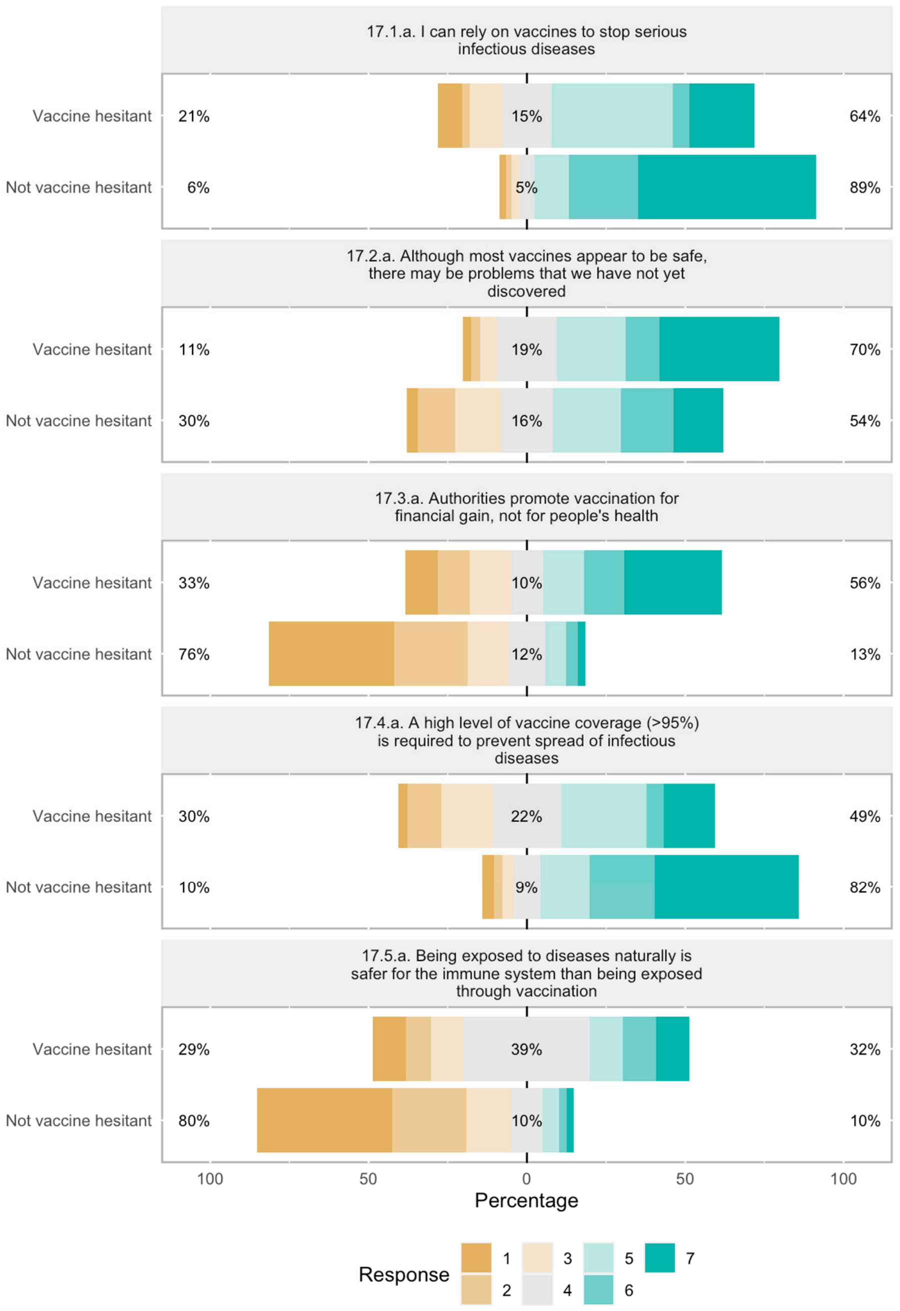

Figure S2. Comparison of vaccine confidence scores for vaccine hesitant and non-hesitant participants. Participants were asked to rank five statements on attitudes to vaccines (the full text of survey question 17 is provided in supplementary information). Ranking was from 1, strongly disagree, to 7, strongly agree. The range of responses for each statement is presented for the hesitant and non-hesitant groups as separate bar graphs with percentages on the left and right indicating disagree (1-3) and agree (5-7) responses, respectively. Note that responses 17.1, 17.2, 17.3 and 17.5 were combined to generate a VAX score.

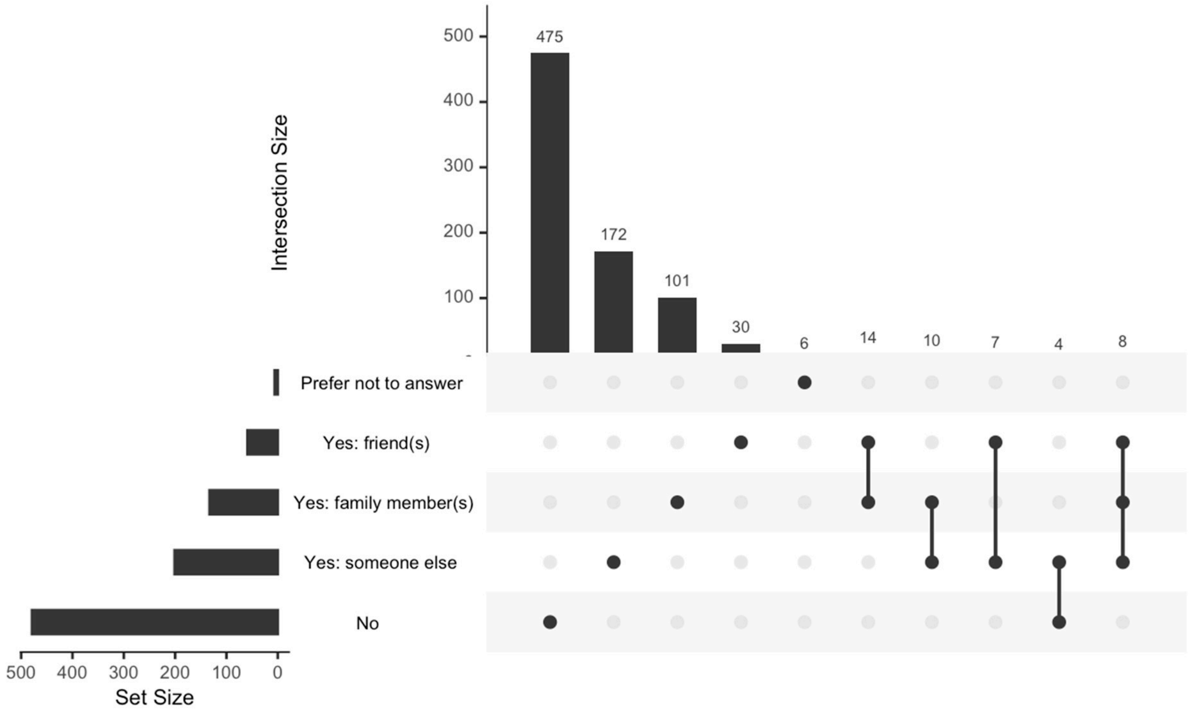

Figure S3. Experiences of deaths among friends, family or others. Question 21 of the survey asked students: "Do you personally know anyone who has died from COVID-19?" Students were allowed to make multiple selections, but in most cases selected one category. Horizontal columns, selection frequency for each individual option; vertical bars, selection frequency for combinations of options.

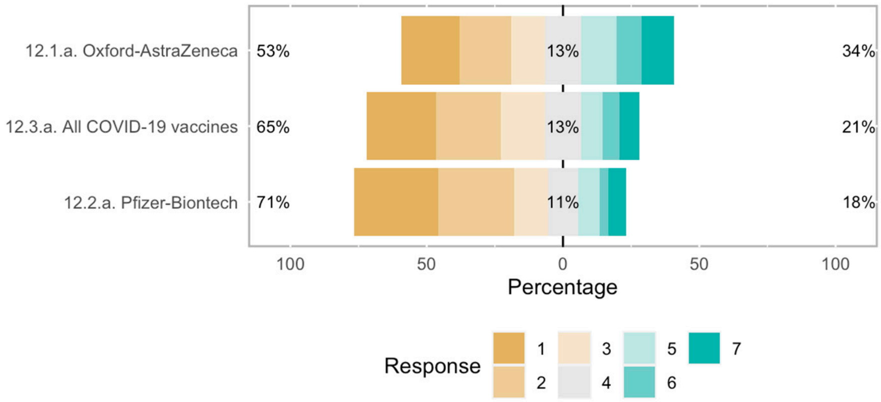

Figure S4. Concerns about the side-effects of COVID-19 vaccines. Question 12 asked participants to indicate the extent to which they agreed with a statement on side-effects (i.e. "I am concerned about the side-effects of COVID-19 vaccines?") for the Oxford AstraZeneca, Pfizer-BioNTech and all COVID-19 vaccines. The extent of agreement was indicated by selecting numbers from 1 (Strongly disagree) to 7 (strongly agree) or Don't know. Percentages for each score are shown in a bar graph with combined percentage values for scores of 1-3, 4 and 5-7 being shown on the left, middle and right, respectively.
